# Presentation and management outcome of tibial infected non-union with Ilizarov technique

**DOI:** 10.12669/pjms.35.1.67

**Published:** 2019

**Authors:** Karim Bakhsh, Atiq-Ur-Rehman, Faridullah Khan Zimri, Eid Mohammad, Wazir Ahmed, Muhammad Saaiq

**Affiliations:** 1 Dr. Karim Bakhsh FCPS, Department of Orthopedics, Civil Hospital, Bolan Medical College, Quetta, Pakistan; 2 Dr. Atiq-Ur-Rehman FCPS, Department of Orthopedics, Civil Hospital, Bolan Medical College, Quetta, Pakistan; 3 Dr. Faridullah Khan Zimri FCPS, Department of Orthopedics, National Institute of Rehabilitation Medicine (NIRM), Islamabad, Pakistan; 4 Dr. Eid Mohammad FCPS, Department of Orthopedics, Civil Hospital, Bolan Medical College, Quetta, Pakistan; 5 Dr. Wazir Ahmed MS, Department of Orthopedics, Civil Hospital, Bolan Medical College, Quetta, Pakistan; 6 Dr. Muhammad Saaiq FCPS, Department of Orthopedics, National Institute of Rehabilitation Medicine (NIRM), Islamabad, Pakistan

**Keywords:** Ilizarov technique, Ilizarov method, Infected non unions of the tibia, Bone healing

## Abstract

**Objective:**

To document the presentation of tibial infected non-union and analyze the management outcome with Ilizarov technique in terms of bone results, functional outcome, bone transport time, external fixation time, external fixation index and any complications.

**Methods:**

This case series was conducted at the Departments of Orthopedic Surgery, National Institute of Rehabilitation Medicine (NIRM), Islamabad and Civil hospital, Quetta over a period of 3-years.

**Results:**

There were 56 patients with 53(94.64%) males and 3(5.35%) females. The age range was 16-50 years with a mean of 32.58±9.98years. According to ASAMI criteria, bone results were excellent in 37(66%), good in 10(17.85%), fair in 6(10.71%) and poor in 3(5.35%). The functional results were excellent in 37(66%), good in 9(16%), fair in 7(12.5%) and poor in 3(5.35%). The bone union rate was 98.21%.

**Conclusion:**

Ilizarov method beautifully addresses the formidable issue of infected non union of tibia with good outcome in terms of bone healing and infection eradication. The treatment period is relatively lengthy and hence patience on part of patient as well as the surgical team is imperative for achieving favourable outcomes.

## INTRODUCTION

Trauma continues to plague the World at large and the low income countries like ours (Pakistan) in particular. It results in frequent mortality as well as high morbidity owing to the associated soft tissue and skeletal injuries. Tibial fractures and subsequent non-unions are not uncommon in the aftermath of such high-energy trauma to the lower limbs. The infected tibial non-unions often pose management challenges to the orthopedic surgeon and his teams. A variety of factors may underlie this problem. For instance, an initial gross contamination of the wound, inadequate initial management, smoking, diabetes mellitus, malnutrition, osteoporosis and any other factors that adversely affect the process of wound healing.^1^-^3^

The tibial non-union may be further complicated by issues such as chronic persistent infection, bone loss, bone gap deformity, limb shortening, and stiff joints. Traditionally a variety of procedures have been employed to address these problems. These include radical debridement, use of antibiotic beads, coverage with different fasciocutaneous flaps or skin grafts, use of bone grafts and vascularized bone flaps. Most often multiple surgical interventions are required that prolong the period of treatment as well as disability.^3^-^5^

Ilizarov technique comprehensively addresses all the aforementioned issues simultaneously. It gives stable fixation and allows immediate weight bearing, ambulation and joint mobility. It allows easy wound management and eradication of infection. It allows filling of the bone gap by promoting bone histogenesis and allowing bone transport. An initial thorough debridement of the infected bone is performed followed by bone transport to fill the residual skeletal defect.^1^,^2^,^6^

The present study was conducted to document the presentation of tibial infected non-union and analyse the management outcome with Ilizarov technique in terms of bone results, functional outcome, bone transport time, external fixation time, external fixation index and any complications encountered during the course of treatment.

## METHODS

This descriptive case series study was carried out at the Departments of Orthopedic Surgery, National Institute of Rehabilitation Medicine (NIRM), Islamabad and Sandeman Provincial Hospital (Civil hospital), Bolan Medical College, Quetta over a period of three years from Jan 01, 2015 to Dec 31, 2017. Informed consent was taken from the patients. The study was conducted after approval from the hospital ethics committees of the hospitals.

It included all patients who presented with infected tibial non-union at the two hospitals and were managed with Ilizarov technique. Our exclusion criteria included patients with polytrauma, associated neurovascular injury and intra-articular injuries.

All the patients were admitted indoor for the definitive management. The initial clinical evaluation was performed with thorough history, complete examination and necessary investigations. Mode of trauma, associated co-morbids such as diabetes mellitus, history of smoking were all sought. Standard X-rays of the affected skeleton were performed among all patients to assess the level and type of non-union, status of the bone, and the type of deformity. Standard wound care and antibiotic therapy was instituted according to tissue culture sensitivity.

The surgeries were undertaken under spinal or general anesthesia. The patient was positioned supine on a radiolucent operating table. The ilizarov external fixator was pre-assembled according to individual requirements of each patient, taking into consideration the limb length and location of non union. The planned incision and the corticotomy sites were marked. The pre-assembled ilizarov external fixator was fixed to the tibial shaft in a standard fashion. Radical debridement of the infected bone and soft tissue was performed. Healthy punctuate bleeding from the bone edges signaled the adequacy of bone debridement. A small incision was made just below the tibial tuberosity to perform a sub-periosteal transverse corticotomy. A sub-periosteal fibulectomy was performed in all cases. Layered closure of the wounds were performed. In case of large wounds, an initial temporization with vacuum assisted closure (VAC) dressings were performed. Any flap coverage required to address the wounds were performed by our plastic surgeon.

The patients were encouraged weight bearing on the first post operative day. Also isometric muscle exercises and range of motion exercises were started. We gave one week latency period before starting distraction. The rate of distraction was 1mm per day, performed as 0.25mm every 6 hourly. Once bone transport was complete the docking ends were compressed by 0.25mm per day until the patient experienced pain at the docking site.

We employed the Association for the Study and Application of Methods of Ilizarov (ASAMI) criteria for evaluating the bone results and functional outcome results in our study.^7^ The ASAMI scoring system for Bone results was as follows:

1. Excellent: Union, no infection, deformity <7°, limb length discrepancy <2.5 cm.
2. Good: Union + any 2 of the absence of infection, <7°deformity and limb length inequality of <2.5 cm.
3. Fair: Union + only one of the absence of infection, deformity <7° and limb length inequality <2.5 cm.
4. Poor: Non-union/ re-fracture/ union + infection + deformity >7° + limb length inequality >2.5 cm

The ASAMI scoring system for the Functional results was as follows:

1. Excellent: Active, no limp, minimum stiffness (loss of <15° knee extension/ <15° dorsiflexion of ankle), no RSD, insignificant pain.
2. Good: Active, with 1 or 2 of the following: Limp, stiffness, RSD, significant pain.
3. Fair: Active, with 3 or all of the following: Limp, stiffness, RSD, significant pain.
4. Poor: Poor inactive (unemployment or inability to perform daily activities because of injury).
5. Failure: Amputation.

The various other outcome measures we recorded were the bone transport time, external fixation time, external fixation index and any complications encountered during the course of treatment. We employed various descriptive statistics to calculate the outcome measures. We used SPSS version 17 (SPSS Inc, Chicago, Illinois, USA).

## RESULTS

We had a total of 56 patients in our study. There were 53(94.64%) males and 3(5.35%) females. Their ages ranged between 16-50 years with a mean of 32.58±9.98 years. Majority of the injuries were secondary to road traffic accidents (n=54;96.42%), followed by fire arm injury and fall from height one each. (n=1;1.78%). Majority of the wounds were closed at the outset (n=30;53.57%), followed by Grade II open (n=17;30.35%) and Grade III open(n=9;16%).

The various bacteria cultured included Staphylococcus aureus 31(55.35%), MRSA 9(16.07%), Escherichia coli 7(12.5%), Pseudomonas aeruginosa 6(10.71%), Klebsiella 2(3.57%), Enterococcus 2(3.57%) and various other bacterial species 12(21.42%). Thirteen of the cultures were polymicrobial whereas the remainder were monobacterial.

History of smoking was present among 27(48.21%) patients. There were 7 (12.5%) patients with diabetes mellitus. Nine (16.07%) patients were malnourished whereas 3(5.35%) patients were obese.

According to ASAMI criteria, bone results were excellent in 37(66%), good in 10(17.85%), fair in 6(10.71%) and poor in 3(5.35%). The functional results were excellent in 37(66%), good in 9(16%), fair in 7(12.5%) and poor in 3(5.35%).

Bone union was achieved among all except one patient. (98.21% bone union rate). The associated soft tissue defects healed by soft tissue transport in 10(17.85%) patients, VAC dressings in 9(16.07%) and fasciocutaneous flaps in 7(12.5%) patients.

The length of the skeletal defect or bone gap ranged from 3 cm to 9 cm with a mean defect of 4.3 cm. The bone transport time ranged from 39-140 days with a mean of 70.10 days. The external fixation time ranged from 5-16 months with a mean of 9.30 months. The external fixation index ranged from 1.2 to 1.6 months/cm with a mean of 1.40 months/cm. Our follow up after fixator removal ranged from 7 months to 36 months with a mean of 20 months.

Among our share of complications included pin tract infection / Shanz-screw site infection among 27(48.21%) patients, K-wires loosening in 7(12.5%), axial deviation during bone transport in 6(10.71%), skin invagination requiring plastic surgical correction in 5(8.92%), flexion contracture of the knee of ≤20^0^ in 2(3.57%), paraesthesia in the distribution of peroneal nerve in 1(1.78%), re-fracture at the docking site after removal of Ilizarov. in 1(1.78%) and persistent infection and amputation 1(1.78%) patient. There were no mortalities in our series.

## DISCUSSION

We employed the Ilizarov technique in our patients with tibial infected non-union cases and had favourable outcome results. The technique was pioneered by the legendry surgeon Ilizarov in 1951. Since then it has earned the status of being the gold standard for the treatment of difficult non-unions of long bones including tibia. The technique entails inserting fine wires percutaneously, tensioning them adequately and attaching to the rings of the rigid circular frame. Hence compression, bone distraction, bone histiogenesis, bone lengthening, and deformity correction are all possible to achieve simultaneously. The fixator is strong and stable, thus allowing ambulation and full weight bearing.^1^,^8^,^9^

Tension stress effect is the fundamental mechanism of the Ilizarov technique. A segmental bone transport is effected after performing a corticotomy in the metaphysis, followed by gradual bone distraction. Ilizarov techniques employ three methods. These include compression osteosynthesis, bone transport and acute compression/ lengthening. The compression osteosynthesis is mainly employed for small defects. The acute compression and lengthening technique is usually considered appropriate for 4-5 cm defects, in order to avoid soft tissue stacking and any neurovascular compromise which can adversely affect the fracture healing. The published literature has reported on successful management of some exceptionally larger size defects in this regard.^10^-^12^ The other commonly used Ilizarov technique is the bone transport which is equally effective for both small as well as very large skeletal defects.^13^-^15^

In all our patients all the wounds were of traumatic origin, mainly road traffic accidents. Among over half the patients, the underlying risk factors for non-unions were smoking and poorly controlled diabetes mellitus. In one published study from UK, half of the patients with such non-unions were active smokers.^16^

In our study, radiological and clinical union was achieved among the majority of cases along with eradication of infection. All of our patients were able to stand and weight bear with partial limb loading immediately after the circular Ilizarov frame was employed. This is regarded as the most essential part of this method. In the published literature, the rate of bone union is reported to be ranging between 87%-100%^4^,^9^ The famous saying of the Ilizarov that “Osteomyelitis burns in the flame of regenerate” beautifully explains the philosophy behind his elegant method of dealing with osteomyelitis. Ilizarov proved that regenerated new bone burns out the bone infection as well as acts as bone graft to fill the bone cavities. He himself never employed any antibiotic or bone graft to treat the bone infection.^17^,^18^

In our study, the mean external fixation index was 1.40 months/cm Our results conform to most of the reported studies where the external fixation index is reported to be in the range of range of 0.55-2.33 months/cm with a mean of 1.46 ± 0.42 months/cm.^4^

In this study, the mean external fixation time was nine months. It favorably conforms to the published studies where it is reported to range from 3.1-13.9 months. With a mean of 9.19 ± 2.22 months.^4^

We employed the ASAMI criteria to evaluate bone and function results among our patients. In the ASAMI criteria, the bone results are based on four criteria namely union, infection, deformity and limb length discrepancy (LLD). The functional results are based on five criteria namely limp, joint stiffness, reflex sympathetic dystrophy, pain and inactivity.^1^,^7^ The ASAMI criteria are easy to comprehend and apply. Moreover these are standardized across the globe.

In our study as per ASAMI criteria, bone results were excellent in 66% patients and good in 17.85%. Our functional results were excellent in 66% whereas good in 16%. Our outcome data conform favorably to most of the published studies the good and excellent rate in functional results ranged from 23%-97% with a mean of 76.03%.^4^,^19^ We had a total of 48 complications. Our share of complications conforms to the reported studies. The mean complications per patient reported in the published literature is 1.47±0.86 with a range of 0.12-3.35.^4^

### Strengths and Limitations of the study

The strengths include the fact that it was conducted in two tertiary care units and included a moderate sample size over a reasonable time period of three years. The limitation of the study is that we did not compare the treatment modality with some other alternative treatment modality. We recommend future multicentre comparative studies to confirm our findings and improve upon our limitations.

## CONCLUSION

Ilizarov method beautifully addresses the formidable issue of infected non union of tibia with good outcome in terms of bone healing and infection eradication. The treatment period is relatively lengthy and hence patience on part of patient as well as the surgical team is imperative for achieving favourable outcomes.

### Authors’ Contributions

FUKZ, KB, AUR & MS: Designed the study and wrote the manuscript.

EM, WA, MS, & AUR: Performed data collection and analysed the results.

All authors have approved the manuscript.
